# The first reported case of furuncular myiasis in Syria with no international travel history of the patient to an endemic area

**DOI:** 10.1093/omcr/omad126

**Published:** 2023-11-28

**Authors:** Jacob Al-Dabbagh, Thaer Douri

**Affiliations:** Faculty of Medicine, Tishreen University, Latakia, Syria; Department of Dermatology, University of Hama, Hama, Syria

**Keywords:** myiasis, infestation, cutaneous myiasis, seborrheic dermatitis

## Abstract

Myiasis is defined as the infestation of any part of the body by fly larvae. It is particularly common in tropical and subtropical regions. Cutaneous myiasis is the most common manifestation of this infestation. Here, we report a 21-year-old Syrian female who presented with a 10-day history of painful 2 ulcer-like lesions on her scalp and was diagnosed with furuncular myiasis, which included more than 20 larvae. The patient had no history of international travel to myiasis-endemic areas before the onset of the lesions. She probably acquired the infestation while visiting a cattle farm located in a rural region east of Hama governorate. Seborrheic dermatitis developed on her scalp after the myiasis treatment was performed.

## INTRODUCTION

Myiasis is defined as the infestation of living humans and vertebrates by larvae of Diptera (flies) that feed and develop on the host’s dead or living tissues [1, 2]. Several dipterous (fly) species can cause myiasis in humans at a diversity of body locations [2]. Skin infestations are the most commonly reported, but myiasis at oral, nasal, aural, ocular, nasopharyngeal, gastrointestinal, pulmonary, and urogenital sites have also been described [2, 3].

Cutaneous myiasis can be classified into three major clinical manifestations: Furuncular, creeping (migratory), and wound (traumatic) myiasis [1, 2, 4]. Furuncular myiasis is characterized as a furuncle-like nodule that develops with one or more larvae within it [3]. The diagnosis of furuncle myiasis is established easily by clinical observation, especially if there has been a travel history to endemic areas [3].

## CASE REPORT

A twenty-one-year-old female presented with a ten-day history of painful nodule-like lesions on her scalp. Before her visit, the lesions were diagnosed as furunculosis by another physician, thus she received antibiotics, which did not lead to improvement.

**Figure 1:**
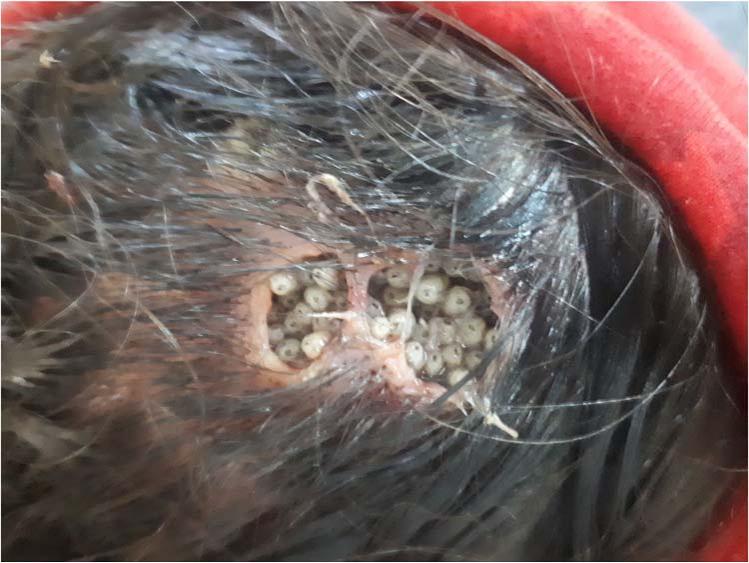
Inspection of the scalp showing two ulcer-like lesions containing numerous larvae.

On examination, two ulcer-like and foul-smelling lesions filled with moving larvae covered by her thick hair were visible to the naked eye on the parietal region. The lesions ranged from 3 to 4 cm in diameter. There was no erythema or edema surrounding the lesions (Fig. 1, Video 1).

The patient did not have proximal lymphadenopathy, fever, or any other systemic symptoms. She had no history of any physical or psychological illnesses/diseases. The patient lives in an urban area in Hama, Syria, and has a medium socioeconomic status. Before the onset of the lesions, the patient recalled insect bites when she visited a cattle farm located in a rural region east of Hama. Based on the clinical examination, the diagnosis of furuncular myiasis was confirmed.

**Figure 2:**
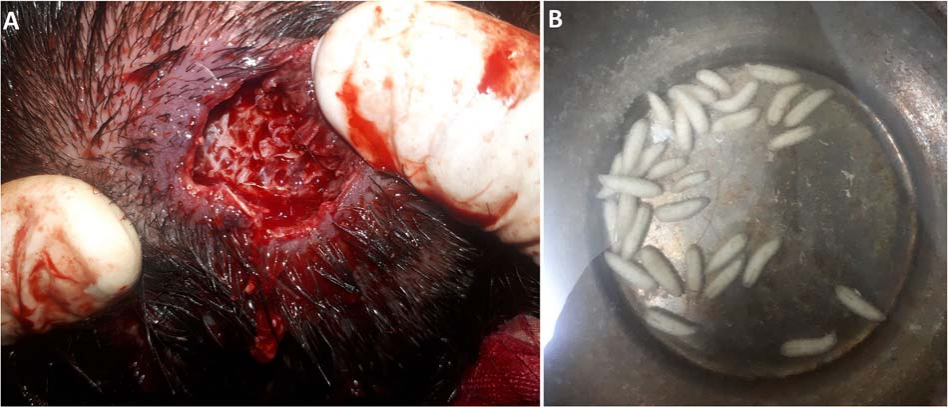
(**A**) The infected lesions after extracting the larvae. (**B**) 30 larvae were extracted from the lesions.

Larvae extraction was performed under general anesthesia on the next day to ensure complete removal of all parasites. Thirty live larvae were extracted from the affected areas. The parasites burrowed into the scalp and reached the periosteum (Fig. 2).

The larvae were collected, but species identification was not possible because of the lack of entomological expertise in the patient’s region. Levofloxacin (750 mg) once daily was initiated and continued for 10 days after the surgical procedure to prevent bacterial superinfection.

**Figure 3:**
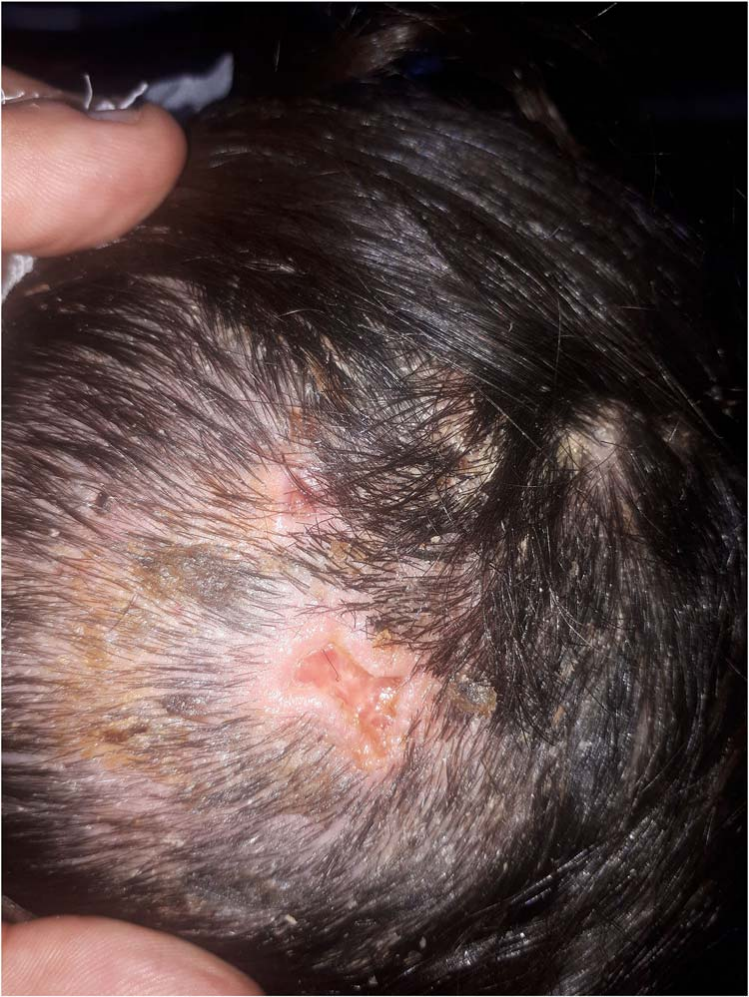
One month later, during the patient’s follow-up, only a minimal alopecic area with scarring was noted at the previous lesions’ location. In addition, seborrheic dermatosis was developed on the scalp.

During the follow-up of the patient, the lesions achieved full recovery after one month. However, the clinical examination revealed erythema and desquamation on the scalp, which suggests the diagnosis of seborrheic dermatitis (Fig. 3).

## DISCUSSION

Furuncular myiasis in humans manifests as a small erythematous papule or nodule that develops into a large and tender boil-like lesion, that has larvae invading the subcutaneous tissue, with a central pore from which serous or purulent fluid discharges [1, 3]. In the central pore, the larval posterior segment that carries the respiratory spiracle may be prominent and visible [3]. However, the lesions in our case had wide apertures through which the larvae were easily visible.

Pain, pruritus, and motion sensation are the most frequently reported symptoms of furuncle myiasis [3]. In this case, the patient complained only of pain.

The fly species that cause furuncular lesions are commonly referred to as ‘bot flies’ [2]. These include Dermatobia hominis, *Wohlfahrtia vigil*, Cordylobia anthropophaga, and the Cuterebra species [4]. Unfortunately, the species of the larvae was not determined in our case.

Although myiasis can occur throughout the world, human botflies are endemic to tropical areas of the New World, which include Mexico, and South and Central America [5, 6]. The majority of cases from developed countries reported in recent years were imported, with the infestation having been acquired during tropical travels [6]. Syria is not included among the areas where myiasis is endemic. Only two cases of myiasis have been reported in Syria in medical journals so far. The first one was an aural myiasis caused by a Lucilia spp and the second one was a subungual myiasis presented as an ingrown toenail, for which no species identification was available [7, 8].

The diagnosis of cutaneous myiasis is usually made clinically and supported by a relevant travel history, but it can be aided by using dermoscopy or imaging procedures such as ultrasound, which help to detect the presence of larvae in the infested tissues and to determine their number [5, 6].

Several agents have been used to treat Dermatobia hominis myiasis in the tropics; these can also be used for furuncular myiasis caused by other fly species [2]. For the treatment of Dermatobia hominis, a triple approach has been proposed: Application of paraffin oil to provoke the larva to extrude; if this is not successful, instillation of lidocaine followed by the removal of the larva with forceps; then finally, surgical removal [9]. Besides the application of paraffin oil, other materials such as occlusive ointment, pork fat, bacon, tobacco, and chewing gum, have reportedly been used in the attempt to suffocate and force the larva to migrate to the surface of the lesion, from where it can easily be extracted [2, 6].

Surgical removal may be necessary if the previous methods fail to draw the larva out of its sinus, or in case of very large and painful lesions, containing several maggots [2]. Moreover, surgical intervention may be preferred because it can ensure the complete removal of the larvae [5].

Cutaneous lesions usually tend to heal rapidly after the larvae have been extracted [2]. Secondary bacterial superinfection has been frequently described as a complication of cutaneous myiasis [2]. In such cases, a short course of proper antibiotic treatment may be advised [2]. In our case, the patient had a surgical intervention to remove all the larvae, followed by 10 days of treatment with levofloxacin.

To our knowledge, furuncular myiasis in association with seborrheic dermatitis has been described in only one case report in the English literature [10]. In our case, seborrheic dermatitis appeared after the patient was treated for myiasis, during the follow-up period.

In conclusion, the diagnosis of furuncular myiasis should be considered in a patient with furuncle-like lesions even if the patient does not reside in or has traveled to epidemic areas. Physicians should consider the possibility of cutaneous myiasis, especially in patients presenting with refractory furunculosis.

This case report hypothesizes that furuncular myiasis could predispose to seborrheic dermatitis. To our knowledge, this is the first case of furuncular myiasis reported in Syria that is published in a medical journal.

## Supplementary Material

video_1_omad126

Supplementary_Video_Legend_omad126

## Data Availability

Data sharing is not applicable to this article as no data were created or analyzed in this study.
