# Supplementary material for: The first reported case of furuncular myiasis in Syria with no international travel history of the patient to an endemic area
Source: Oxf Med Case Reports. 2023 Nov 28;2023(11):omad126. doi: 10.1093/omcr/omad126 (PMC10686010; doi:10.1093/omcr/omad126)
Supplement: Supplementary_Video_Legend_omad126 [file supplementary_video_legend_omad126.docx]

Video 1: Numerous moving larvae, which are visible to the naked eye.
